# Home sweet home: spatiotemporal distribution and site fidelity of the reef manta ray (*Mobula alfredi*) in Dungonab Bay, Sudan

**DOI:** 10.1186/s40462-022-00314-9

**Published:** 2022-04-28

**Authors:** Anna M. Knochel, Nigel E. Hussey, Steven T. Kessel, Camrin D. Braun, Jesse E. M. Cochran, Graham Hill, Rebecca Klaus, Tarik Checkchak, Nasereldin M. Elamin El Hassen, Mohammed Younnis, Michael L. Berumen

**Affiliations:** 1 Red Sea Research Center, Division of Biological and Environmental Science and Engineering, King Abdullah University of Science and Technology, Thuwal, 23955 Kingdom of Saudi Arabia; 2 Department of Integrative Biology, University of Windsor, 401 Sunset Avenue, Windsor, ON Canada; 3 Equipe Cousteau, Paris, France; 4 Daniel P. Haerther Center for Conservation and Research, John G. Shedd Aquarium, Chicago, IL 60605 USA; 5 Biology Department, Woods Hole Oceanographic Institution, Woods Hole, MA 02543 USA; 6 The Deep Aquarium, Hull, UK; 7 Wildlife Conservation General Administration, Port Sudan, Sudan

**Keywords:** *Mobula alfredi*, Movement ecology, Sudan, Acoustic telemetry, Conservation, Red Sea

## Abstract

**Background:**

Reef manta ray (*Mobula alfredi*) populations along the Northeastern African coastline are poorly studied. Identifying critical habitats for this species is essential for future research and conservation efforts. Dungonab Bay and Mukkawar Island National Park (DMNP), a component of a UNESCO World Heritage Site in Sudan, hosts the largest known *M. alfredi* aggregation in the Red Sea.

**Methods:**

A total of 19 individuals were tagged using surgically implanted acoustic tags and tracked within DMNP on an array of 15 strategically placed acoustic receivers in addition to two offshore receivers. Two of these acoustically monitored *M. alfredi* were also equipped with satellite linked archival tags and one individual was fitted with a satellite transmitting tag. Together, these data are used to describe approximately two years of residency and seasonal shifts in habitat use.

**Results:**

Tagged individuals were detected within the array on 96% of monitored days and recorded an average residence index of 0.39 across all receivers. Detections were recorded throughout the year, though some individuals were absent from the receiver array for weeks or months at a time, and generalized additive mixed models showed a clear seasonal pattern in presence with the highest probabilities of detection occurring in boreal fall. The models indicated that *M. alfredi* presence was highly correlated with increasing chlorophyll-a levels and weakly correlated with the full moon. Modeled biological factors, including sex and wingspan, had no influence on animal presence. Despite the high residency suggested by acoustic telemetry, satellite tag data and offshore acoustic detections in Sanganeb Atoll and Suedi Pass recorded individuals moving up to 125 km from the Bay. However, all these individuals were subsequently detected in the Bay, suggesting a strong degree of site fidelity at this location.

**Conclusions:**

The current study adds to growing evidence that *M. alfredi* are highly resident and site-attached to coastal bays and lagoons but display seasonal shifts in habitat use that are likely driven by resource availability. This information can be used to assist in managing and supporting sustainable ecotourism within the DMNP, part of a recently designated UNESCO World Heritage Site.

**Supplementary Information:**

The online version contains supplementary material available at 10.1186/s40462-022-00314-9.

## Background

The movement behavior of large marine vertebrates is strongly impacted by habitat complexity rather than evolutionary origin across a wide range of taxa [1]. Movements can be shaped by foraging opportunities and reproductive ecology [2, 3], predator avoidance [4, 5], and environmental needs [6], all of which are influenced by scale-dependent environmental factors. As anthropogenic impacts continue to increase in coastal and open ocean ecosystems, describing patterns of megafauna movements [7] and identifying the ecological, physiological, and oceanographic drivers of those movements is a priority [8]. Understanding species’ habitat selection will likely by critical to the conservation of these species in the face on anthropogenic change.

The reef manta ray (*Mobula alfredi*) is a large, reef-associated, filter-feeding batoid that is widely distributed in tropical and subtropical regions of the Indo-Pacific [9]. While capable of long-distance movements spanning hundreds of kilometers [10–12], the species is commonly found in shallow coastal and lagoonal habitats [13–15]. *Mobula alfredi* abundance and habitat selection within these areas is often seasonal [16–20], most likely in response to predictable fluctuations in food availability and distribution [21]. Due to their preference for near-shore habitats, *M. alfredi* are regularly exposed to human activities and have been heavily fished in several regions [22], both directly for their gill plates and indirectly as bycatch. As a highly k-selected elasmobranch (i.e. one pup per litter; [23, 24]) with a very low maximum intrinsic population growth rate [25], *M. alfredi* populations are at risk of rapid depletion and local extirpation in regions of sustained targeted fishing [26]. As a result, *M. alfredi* is officially classified as “Vulnerable” by the International Union for the Conservation of Nature (IUCN) due to declines in abundance reported from several known aggregation sites [27].

In Mozambique, models indicate that *M. alfredi* sightings declined by 88% over an eight-year period [26] which is thought to have been caused by increased fishing pressure [28]. Other *M. alfredi* cohorts along the eastern coast of Africa (Somalia, Tanzania, Kenya, Madagascar) are understudied but are also thought to be threatened by human activities [27]. One possible exception is the Dungonab Bay and Mukkawar Island National Park (DMNP), located on the Northeastern Sudanese coast. The DMNP hosts the largest known *M. alfredi* aggregation in the Red Sea [27, 29] and is considered a globally important site for the species’ conservation [30]. While individuals are incidentally captured in artisanal gill net fisheries in the vicinity [30] there does not appear to be a regionally active fishery for *M. alfredi* or other devil rays [31]. DMNP may serve as a key refuge or source population for *M. alfredi* in the Red Sea and the broader East African coastline, but the population dynamics and movement ecology of *M. alfredi in* this region remain understudied.

Tracking marine megafauna movement patterns is complex, but with rapid technological advances in telemetry approaches, the monitoring of individual animals is now possible over a wide range of temporal and spatial scales, from examining local habitat preferences [15] to ocean-spanning migrations [32–34]. Passive acoustic telemetry, which employs a network of receivers to record semi-continuous presence/absence data of tagged individuals, offers long-term monitoring of individuals but is limited by spatial coverage of receivers. By contrast, smart positioning satellite-linked tags (SPOTs) can provide accurate near real time positional data of an animal independent of fixed receivers, but this technology is dependent on animals exhibiting regular surfacing behavior. When used in a dual tagging approach, these techniques can provide a detailed understanding of the spatio-temporal movements and habitat use of individuals, including residency patterns, core habitat use, and larger scale movement ecology [34–36]. Moreover, satellite linked tags can reveal movements of acoustically tagged individuals when they move outside the array of fixed receivers or when array design is discontinuous and consequently animals can be present in a region but not detected [37]. Pop-off satellite archival tags (PSATs) can also be used to map broad-scale movements through the measurement of light levels and geolocation modeling, but resulting movement estimates are often characterized by significant uncertainty [38, 39]. However, a dual tagging approach utilizing both PSAT and acoustic tags allows for geolocation models to incorporate “known” acoustic locations to better constrain horizontal track estimates [36].

Here, acoustic and satellite telemetry data (SPOT and PSAT) is used to quantify *M. alfredi* movements within the DMNP and the surrounding region. We characterize seasonal presence/absence of individual*s* within the Bay, describe long-distance movements, identify high-use areas, and site fidelity. Acoustic data were analyzed in conjunction with biotic (sex and maturity) and abiotic (chlorophyll-*a* and lunar illumination) parameters to identify potential drivers of observed behaviors. Results of *M. alfredi* movements within DMNP are discussed in the context of current knowledge on global reef manta ray movements and relative to local and regional conservation efforts and priorities.

## Materials and methods

### Study site

**Fig. 1:**
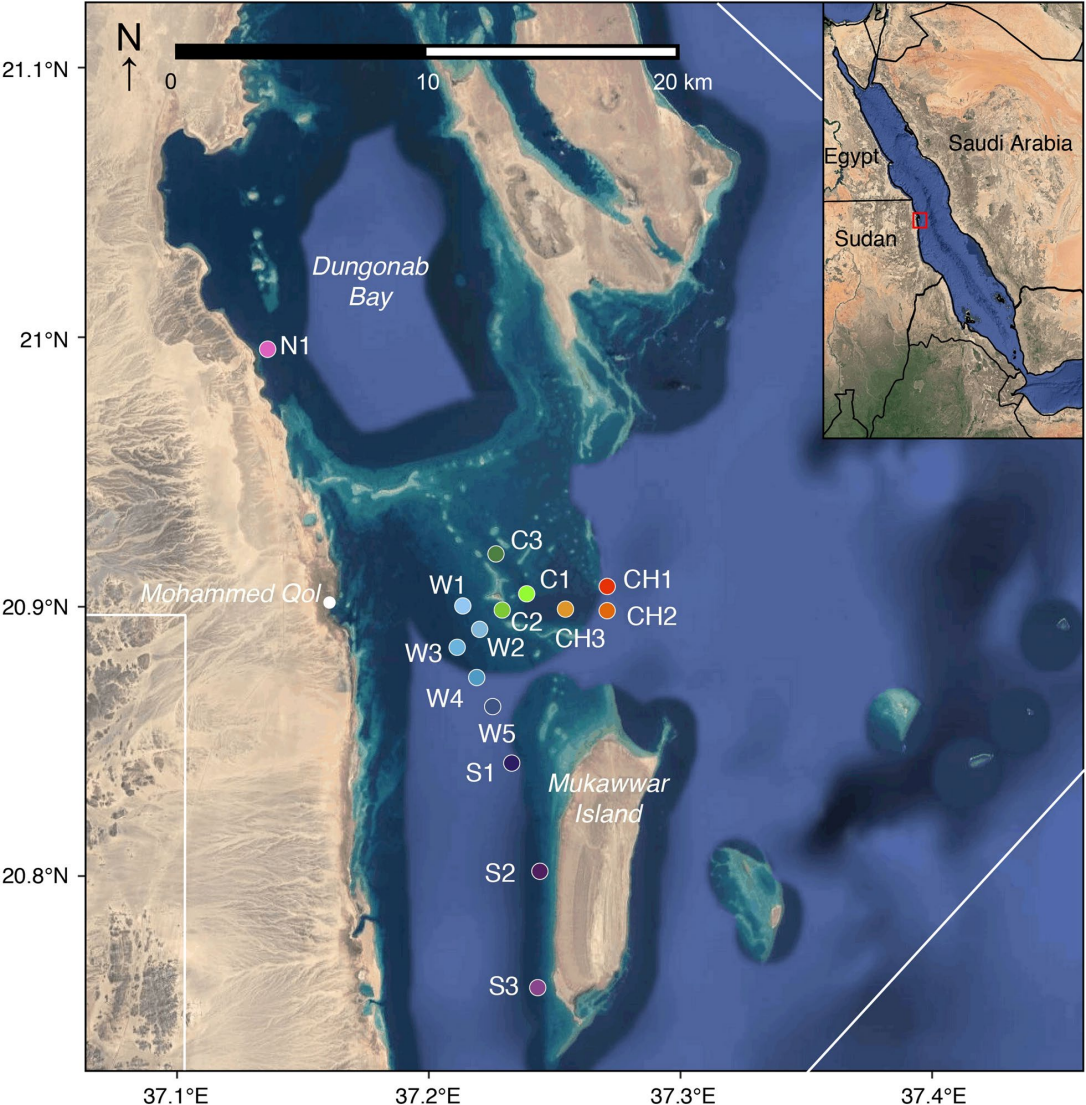
Location of individual receivers in the Dungonab Bay and Mukkawar Island National Park acoustic array in Dungonab Bay, Sudan used to detect tagged *Mobula alfredi*. All receivers were placed a minimum distance of one kilometer apart. The two furthest receivers (N1 and S3) were ~ 29 km apart. White lines indicate the border of the MPA. Color coding is based on the geographic position of the receivers as described in the methods: (i) CH = Channel (reds); (ii) C = Central (greens); (iii) W = West (blues); (iv) S = South (purples); (v) N = North (pink)

The Dungonab Bay and Mukkawar Island National Park (DMNP) is one of two legally declared marine protected areas in Sudan located along the Red Sea coast of northern Sudan (Fig. 1, 20° 52′ N, 37° 14′ E). The area is principally used by local semi-commercial, small-scale fisheries, though it has also been exploited by Egyptian fishing boats [40–42]. The use of gillnets by some fishermen within DMNP (NEH personal observation) poses a potential threat because the gear is indiscriminate and occasionally results in *M. alfredi* bycatches. Other human impacts on the DMNP are otherwise limited to tourism activities via liveaboard diving boats, originating from both Egypt and Sudan, and light boat traffic from the nearby villages [40–42]. The area was designated as a National Park in 2004 and was the first marine protected area (MPA) in the Red Sea to be added as a serial site to the UNESCO World Heritage List in 2016, together with Sanganeb Atoll Marine National Park [43].

### Receiver array

The main array consisted of 15 Vemco VR2W-69 kHz acoustic receivers equipped with lithium-ion batteries (Fig. 1). Two additional receivers were deployed south of the Bay in two areas of interest (Suedi Pass and Sanganeb Atoll) in order to detect migration between these sites (Additional file 1: Fig. S1). Station locations were chosen based on *M. alfredi* occurrence and aggregation behavior gathered from historical data [29], local Bedouin traditional ecological knowledge, regional reef surveys and liveaboard dive operator experience. This combined knowledge resulted in the placement of receivers in five core regions: (i) within the main entrance channel into DMNP that connects the shallow reef area to deeper water outside the Bay (n = 3); (ii) in an area of shallow patch reefs at the end of the channel, a known site where *M. alfredi* are observed at the surface during the fall period, (n = 3); (iii) on the edge of the southern basin (n = 5), (iv) along the western side of Mukkawar Island (n = 3); and (v) in a nearshore site close to the local village of Mohammed Qol (n = 1; identified by fishers’ traditional ecological knowledge). Receivers (n = 15) were either attached to a riser with a sub-surface float (positioned ~ 1 m below the float using zip ties and security string) that was then connected to chain or rope covered with hosepipe and secured through reef structures or were attached to rebar sand anchors screwed into the seabed. Within the DMNP, receivers were spaced at least 1 km apart within the core sections with the longest direct distance between the two furthest receivers, S3 and N1, equal to 29 km. Range testing was not conducted in-situ. Thus, receivers were spaced at least 1 km apart using an assumed 50% detection range of 540 m based on range tests conducted in similar Red Sea reef environments [44].

### Animal tagging

Between October 28th and November 1st^t^ 2012, *M. alfredi* (n = 20) were captured and tagged using a modified hook and line approach [30]. Specifically, free swimming *M. alfredi* were approached slowly using a small fiberglass tender with a guide directing the vessel towards either the left or right side of the animal, while avoiding direct contact. A breakaway rig was then used to capture the animals. The rig consisted of a 20/0 circle hook attached to a 30 m length of 1.5 cm diameter twisted nylon rope, with two 20-L plastic containers tied to the other end. The 20/0 hook rig was attached with a break away link to the end of a 2 m tagging pole.

On locating the animal, the guide maneuvered the tagging pole over the head of the animal and placed the hook in the center of the palatoquadrate. Hooked individuals were allowed to swim towing the rig for about 15 min until fatigued. The individual was then maneuvered to the side of the boat and a tail rope was placed over the tail and fastened around the dorsal fin. VEMCO V16-6H acoustic transmitters (N = 20; nominal delay range: 310 to 410 s) were surgically implanted in the peritoneal cavity through a ~ 5 cm long incision, and the incision was closed with three to four interrupted sutures. Sex was determined based on the presence or absence of claspers and wingspan was measured directly in the water to the nearest centimeter. Individuals were divided into categories of immature and mature based on clasper calcification for males (calcification indicating maturity) and known sizes of maturity for females (a disc width of 3.2 m or greater indicating maturity [23]).

Four acoustically tagged mantas were also fitted with external satellite transmitters, including one (M13) Wildlife Computers SPOT5 tag (a type of tag that transmits horizontal position data to a satellite when the animal surfaces) and three (M20, M21, M22) Wildlife Computers MK10AF tags with Fastloc GPS capability (a type of PSAT that records light levels for geolocation models and can transmit Fastloc GPS and Argos derived position points when the animal surfaces). The SPOT tag was mounted onto the dorsal fin via four nylon bolts and associated lock nuts through four holes predrilled using a handheld electric drill [30], while the MK10AF tags were anchored using a large plastic Doemier dart attached to ~ 10 cm leader in the dorsal musculature located on posterior right section of the body.

### Residency

To filter out echoes, signal collisions, and other sources of detection error, isolated single acoustic detections were first removed from the DMNP dataset [45]. To be considered present in the array on a given day, two detections of an individual were required within that same calendar day. Individual detections were then eliminated if two subsequent detections between receivers resulted in unrealistic rates of movement. A swim speed of > 2 m/s was selected to filter these data, based on previous estimates for mobula rays [46–48]. To avoid analyzing unnatural movements associated with the capture-tagging process, detections recorded within 48 h post release were excluded. The filtered data was then used to calculate an individual maximum residency index (RI) equal to the number of days a tagged manta ray was detected within the DMNP array divided by the number of days between the date of first and last detection [18]. Shapiro Wilk and Levene’s Test revealed RI data were normally distributed but variance was heterogeneous; therefore, residency indices were tested for significant differences between males and females and within size classes with Welch’s T-Test and a Pearson’s correlation, respectively.

### Modeling presence

The influence of biotic and abiotic parameters on *M. alfredi* presence within the DMNP was tested using generalized additive mixed-effects models (GAMMs) with the ‘mgcv’ package [49] in R version 3.6.3 [50]. All filtered detections were included in the models, despite interspersed receiver battery failure that occurred in the last four months of the study. This variation in receiver effort due to random battery failure was accounted for by including the number of active receivers for each hour as a fixed variable in the tested models. Acoustic detections were incorporated as a binomial response variable of hourly presence with each individual labelled with a value of “1” if the animal was detected during that hour and a value of “0” if not. To examine trends in short and long-term habitat use, hour of the day and day of the year were included as cubic cyclical smoothing parameters. To assess how the biological traits of *M. alfredi* affected patterns of presence in DMNP, sex (male/female) and maturity status (immature/mature) were incorporated as fixed variables in the models.

Variables such as chlorophyll-*a* concentration and lunar phase are known to influence *M. alfredi* movement patterns [15, 18, 21] and were included in the models. Remotely sensed chlorophyll-*a* was used to provide a reasonable proxy for ocean productivity [21]. Interpolated remotely sensed daily chlorophyll-*a* concentration (mg/m^3^) data was obtained from E.U. Copernicus Marine Service Information at a 4 km^2^ resolution block centered over the central DMNP array. Lunar phase was quantified as the fraction of the moon illuminated and was obtained through the United States Naval Observatory. Lastly, individual *M. alfredi* ID was included as a random effect in all tested models. While other factors, such as current strength, tidal flux, and local wind speeds are known to affect *M. alfredi* movement ecology [15, 18], these were either not available or could not be collected in DMNP due to the logistics of working in a remote region. Models were constructed for combinations of the smoothed terms: Day of year, Hour, chlorophyll-*a*, and lunar illumination (Table 1), resulting in the testing and comparison of 16 models. Model selection was based on the Akaike Information Criterion (AIC) with the lowest AIC score indicating the most parsimonious model (Additional file 2: Table S1).

**Table 1:** A summary of variables tested in the General Additive Mixed Models (GAMMs) including day of year, time of day, the fraction of moon illuminated, and Chlorophyll-*a* concentration

Variable	Resolution	Units	Spline
s(Day)	Daily	1–365	Continuous; cubic cyclical, k = 7
s(Hour)	Hourly	0–23	Continuous; cubic cyclical, k = 7
s(Moon)	Daily	0.00–1.00	Continuous; k = 6
s(CHLA)	Daily	0.01	Continuous; k = 7
s(MANTAID)	NA	NA	Fixed; Random effect
Sex	NA	Female/Male	Fixed
Maturity	NA	Immature/Mature	Fixed
nStations	Daily	3–15	Fixed

The models were tested using the individual identity of *Mobula alfredi* as a random effect in addition to two demographic variables (sex and maturity state) and the number of active receivers (nStations) to account for varying receiver effort due to battery failure

### Receiver visitation and movements

Visitation patterns to individual receivers were quantified via residency and non-residency events using the ‘Vtrack’ package in R [51]. A residency event was triggered after two subsequent detections at a receiver and ended when either the individual was detected at a different receiver or an hour-long period elapsed without any further detections [52]. Non-residence events were defined as periods of complete detection absence from the DMNP array and were calculated to examine the longest period between detections for each individual (maximum non-residence). The total minimum distance moved (i.e. direct straight-line movements between individual receivers) were summed for each individual for each day and over the entire study period. To visualize movements and connectivity within the DMNP array, detection data formatted in ‘Vtrack’ [51] were used to create networks representing movements among all receivers in ‘igraph’ [53] and visualized in ‘ggplot2’ [54]. Within the networks, nodes are represented by each receiver with their relative size indicating the total number of detections at each given receiver. Edges were weighted by the number of movements between receivers, which are assumed to represent subsequent detections or repeat visits between receivers.

### Spatial distributions

The raw detection record contains discrete spatial data, specifically the known position of the detecting receiver. To convert these data into more continuous estimates of animal location, each manta’s detection record was grouped into 6-h bins and used to calculate mean centers of activity (COAs) for those periods. COA analysis was performed using the Animal Tracking Toolbox (ATT) [55] in the ‘adehabitatHR’ package of R [56]. A timestep of 6 h (360 min) was chosen after initially testing timesteps of 60, 120, 180, 360, and 720 min. To assess seasonal shifts in spatial activity within the DMNP array, COAs from all individuals were pooled and kernel utilization distributions (KUDs) were calculated for each month at 50% and 95% levels using a reference-bandwidth (href) smoothing parameter.

Satellite tag data (PSATs, model MK10AF) were decoded using tag manufacturer software and most probable track estimates were constructed using a proprietary hidden Markov model framework (WC-GPE3, Wildlife Computers) [57]. The model computes gridded posterior probability distributions to estimate the most likely state (position) at each time point using light-level, sea surface temperature (SST), and bathymetric constraints. Tag-based observations were compared to NOAA’s 1/4° daily Optimum Interpolation Sea Surface Temperature (OISST) product and bathymetric constraints were implemented relative to ETOPO1 [58]. The speed parameter in the model was fixed a priori at 2 m/s and was used to build daily diffusion kernels that are convolved with light and SST-based likelihoods on a 0.25° grid. This method has been shown to reliably reconstruct large-scale animal movements for surface-oriented species with quality light and SST data (i.e. to within ~ 80 to 150 km root-mean-square errors [59]), although similar approaches have resulted in significant uncertainty in movement estimates in restricted basins such as the Red Sea [38].The dual-tagging approach yielded known acoustic telemetry derived positions that were treated as “fixed” locations in the geolocation model. These incorporated acoustic telemetry locations were calculated using COAs based on a 720 min (12 h) timestep.

Similarly, Argos satellite positions from a single SPOT tagged individual were pooled with the ‘Vtrack’ acoustic telemetry visitation data (see above). This allowed for absences from the acoustic array to be correlated to any movement recorded by the SPOT tag.

Detections of *M. alfredi* on receivers located outside the main DMNP array were evaluated independently from the above analyses to qualitatively describe larger scale movements and validate absence from the DMNP array. This was undertaken due to the small number of detections on these external receivers (n = 5). While three of these events consisted of only single detections, the far lower densities of tagged individuals at these sites made signal collisions and false detections less likely to occur. Isolated detections were therefore considered valid for the offshore receivers.

## Results

### Detection summary and residency

From November 2nd, 2012 to October 24th, 2014, a total of 52,909 acoustic detections of *M. alfredi* were recorded across the main DMNP array. After removing isolated single records (from the main DMNP array; n = 710), detections within 48 h of tagging (n = 102), and unrealistic detections based on speed between receivers (n = 666), the analyzed dataset consisted of 51,431 detections of 19 tagged *M. alfredi* (9 females, 10 males) for a mean of 2707 ± 1802 SD detections per individual. Tag retention was high; fifteen individuals were tracked for more than 690 days during the 722-day study. Maximum track durations were limited by receiver battery life (with only one deployment possible due to the geopolitical situation in Sudan), rather than transmitter battery life or potential departure from the site. The remaining four individuals were tracked from 122 to 527 days. The single individual for which 77 detections were recorded may have shed the tag prematurely because of improper tag placement and thus was excluded from further analysis. *Mobula alfredi* were detected throughout the year with the DMNP array recording at least one tagged animal on 695 out of 722 days (96% of days between the first detection to last detection of the collective group of *M. alfredi*). The maximum number of *M. alfredi* recorded across the DMNP array on any given day was 17, which occurred five times during the study and always during the boreal fall. Individuals spent considerable portions of their time within the DMNP array; Residence Index values ranged from 0.17 to 0.62 (Table 2) with an average of 0.39 ± 0.13 SD. Despite high residence, some animals were not detected by the DMNP array for extended periods of up to 149 days with an average individual maximum mean of 53.9 ± 30.9 SD days between subsequent detections. Females recorded a greater number of total detections (female = 30,759; male = 20,672), greater average detections per individual (female = 3418; male = 2067), and a higher maximum RI (female = 0.41; male = 0.39) than males, but the difference in RI between the sexes was not significant (Welch’s T-Test, p_RI_ = 0.61). Detection counts and RI showed negligible correlation with size (r = − 0.24 and r = 0.04, respectively). RI was also similar between mature (n = 15) and immature individuals (n = 4, Welch’s T-Test with homogenous variance, p_RI_ = 0.89).

**Table 2:** Summary of acoustic detections for manta rays *Mobula alfredi* in Dungonab Bay, Sudan spanning November 2, 2012 to October 24, 2014

Manta ID	Size (cm)	Sex	Maturity	Deployment date	Total detections	Track days	Detection days	RI	Minimum distance traveled (km)	Max consecutive days of absence
M3	308	M	Mature	10/28/12	2782	702	352	0.50	1554.0	35
M6	326	F	Mature	10/29/12	2701	709	283	0.40	1032.3	46
M7	304	M	Mature	10/29/12	881	699	134	0.19	493.5	80
M8	325	F	Mature	10/29/12	2491	710	259	0.36	1126.5	56
M9	326	M	Mature	10/29/12	2621	715	316	0.44	1362.0	25
M10	246	F	Immature	10/30/12	8659	722	383	0.53	2771.8	96
M11	272	M	Immature	10/30/12	124	122	21	0.17	73.7	48
M12	282	M	Immature	10/30/12	2172	700	220	0.31	991.0	60
*M13	366	F	Mature	10/30/12	1955	527	188	0.36	736.5	37
M15	264	M	Immature	10/30/12	4063	703	393	0.56	1811.0	29
M16	346	F	Mature	10/30/12	3270	711	300	0.42	1174.4	32
M17	344	F	Mature	10/31/12	2463	714	255	0.36	1006.9	71
M18	316	M	Mature	10/31/12	2786	712	308	0.43	1128.8	24
*M20	296	M	Mature	10/31/12	1032	707	125	0.18	483.1	149
*M21	320	F	Mature	10/31/12	1739	691	196	0.28	673.3	71
M22	344	F	Mature	10/31/12	3280	718	276	0.38	1321.8	32
M23	362	F	Mature	11/1/12	4201	715	368	0.51	1596.9	22
M24	314	M	Mature	11/1/12	3298	431	268	0.62	1298.7	58
M25	316	M	Mature	11/1/12	913	265	79	0.30	412.8	53

“Track Days” is a measure of each animal’s detection period from the day of the first detection to the day of the last detection. “RI” stands for residence index and is calculated as the number of days detected in the array divided by the number of days between first and last detection. Individuals simultaneously fitted with a satellite tag are marked by an asterisk (*)

### Drivers of presence

Detection records were converted into 329,061 hourly binomial observations of *M. alfredi* presence/absence. These data were then used to fit 16 candidate GAMMs (Additional file 2: Table S1). The selected model with the lowest AIC explained 10.9% of the total variance and revealed significant seasonal (*p* < 0.001) and diel (*p* < 0.001) trends and significant correlations between *M. alfredi* presence and chlorophyll-*a* (*p* < 0.001) and lunar illumination (*p* < 0.001). The model incorporated the maturity and sex of tagged *M. alfredi* as well as fluctuations in receiver effort. Neither maturity (*p* = 0.888) nor sex (*p* = 0.135) showed a significant influence on detection probability in the chosen model. Increasing receiver effort resulted in higher detection probabilities in the model (*p* < 0.001).

**Fig. 2:**
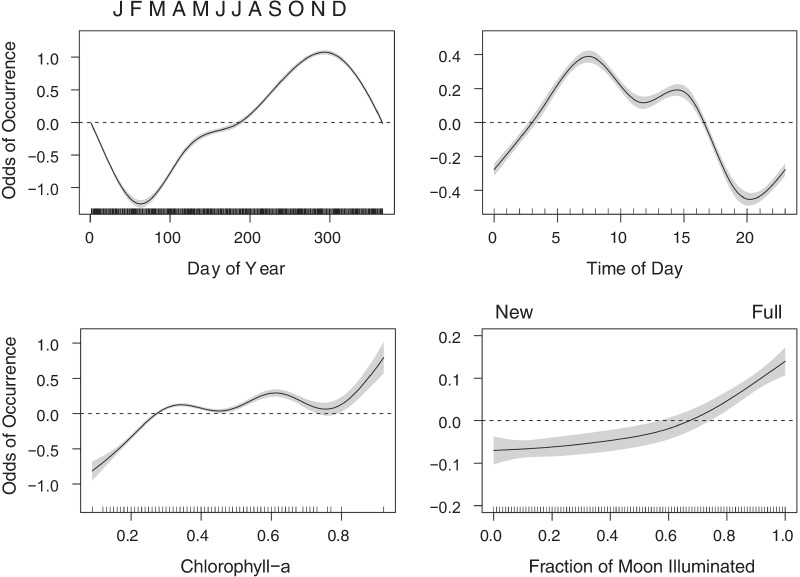
GAMM graphical outputs for each variable included in the selected model to determine their influence on the probability of *Mobula alfredi* presence in Dungonab Bay from November 2, 2012 to October 24, 2014. Variables included day of year, hour of day, chlorophyll-*a* (as a proxy for productivity), and fraction of moon illuminated. The degree of the effect is represented by the magnitude of the y-axis

The selected model was used to explore the effect of different parameters on the probability of *M. alfredi* presence in DMNP (Fig. 2). Seasonal variability in detection probability ranged from a low of approximately 20% in February and March to a maximum of 75% in October. Detection probability also varied on hourly timescales, peaking in the early morning and afternoon between 5:00 and 10:00 and at 15:00 respectively, then declining throughout the rest of the evening and reaching a minimum around 20:00 before increasing through the night until the next morning’s peak. The model showed that detection probability increased with increasing concentrations of chlorophyll and with increased lunar illumination.

### Movement within the array

**Fig. 3:**
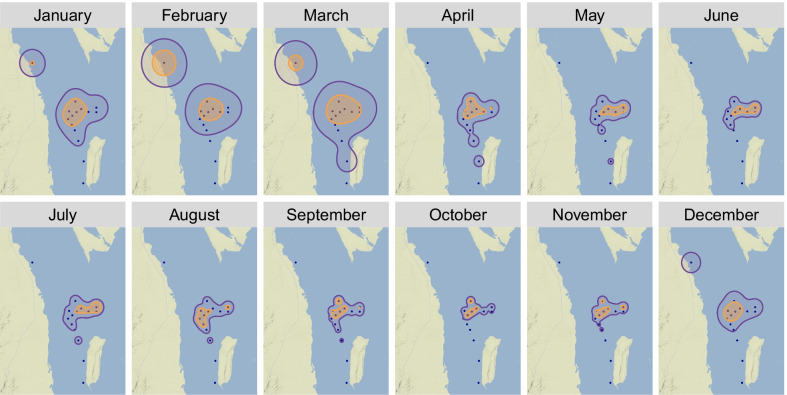
Spatiotemporal distribution of acoustic detections of *Mobula alfredi* in Dungonab Bay represented by 50% (orange) and 95% (purple) monthly kernel utilization distributions. Black dots indicate receiver locations

Hotspots of activity occurred near the mouth of the channel to DMNP in May, June, and July, and shifted inwards to the central part of the DMNP array in September, October, and November (Fig. 3). Home range estimates (95%) within the DMNP array were largest between December and March and smallest between April and November (Fig. 3). Detections were only recorded on the northmost receiver (N1) between November and March of both years, coinciding with gaps in detections for most other receivers. Of the 19 tagged individuals, 17 were recorded making this movement to N1 at some point during those months. Activity at this station peaked in February, which accounted for nearly half of all detections within the acoustic array during this month. Similarly, *M. alfredi* were only detected on the far southern receiver (S2) between March and May. These patterns were corroborated by both the aggregate monthly KUDs, which expanded both to the north and to the south during the months of December to March (Fig. 3) and by the individual detection records which showed a predictable seasonal cycle of regional habitat preferences.

The mean receiver residency period obtained through ‘Vtrack’ for each individual was 37.15 min; however, the standard deviation was high (± 50 min) indicating wide variation in residency duration. The longest residency event occurred at the north-central station (C3) in June where an individual was detected 67 times over a nine-hour period. There were 262 individual residency events that lasted > 3 h that were recorded at 12 of the 15 receivers, showing that these sites were frequently used. The northern channel (CH1) recorded the highest number of these events (n = 93) followed by C3 (n = 53). These long receiver residency events were also seasonally distributed and followed the same temporal trends of hourly detections identified through the GAMMs. Records of *M. alfredi* spending > 3 h at various receivers were highest in June, September, and November, and lowest from February to April.

**Fig. 4:**
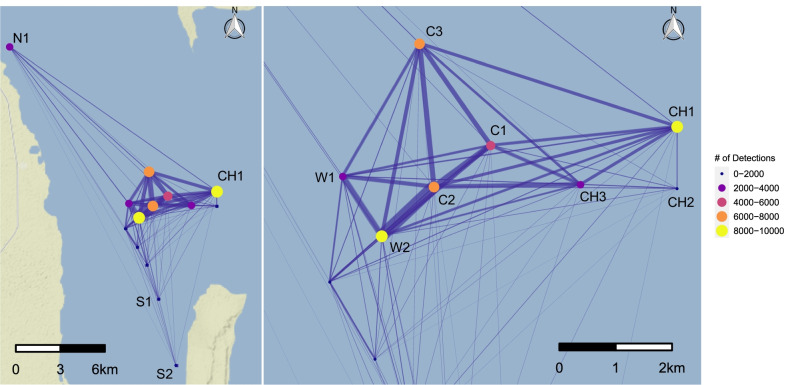
Movement network of *Mobula alfredi* in Dungonab Bay. Nodes represent individual acoustic receivers with the size of the node proportional to the total number of detections. Edge thickness represents the frequency of movements between two receivers. Right panel depicts a closer view of movement networks in the central section of the DMNP array. The most southern station S3 did not record detections and was removed for mapping purposes

Individuals moved a minimum average of 5.76 km and a maximum of 51.2 km per day within the DMNP array. Movements between receivers were highest between C2 and W2 (n = 828) and between W1 and W2 (n = 371) (Fig. 4). The majority of detections occurred at station CH1 (n = 9,082, 17.7% of total detections), located on the north corner of the entrance to the channel. No detections were recorded on the far southern receiver S3 located close to the western shoreline of Mukkawar Island. Of the four individuals whose detections ceased before the end of the study, three were last recorded at station CH1 located at the mouth of the channel entrance to DMNP.

### Broad-scale movement

**Fig. 5:**
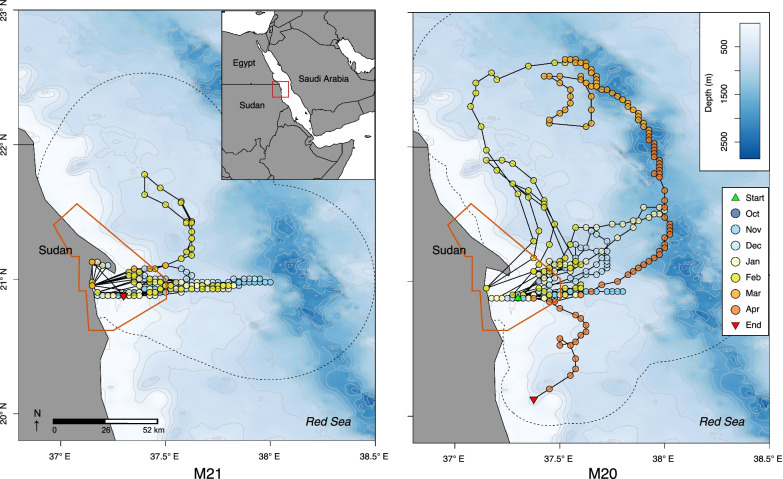
Spatial–temporal residency duration plot for an adult female *Mobula alfredi* (M13), equipped with both a SPOT and acoustic tag. Greyscale points represent transmissions from the SPOT5 tag and their estimated accuracy errors (3: < 250 m; 2: 250 to < 500 m; 1: 500 to < 1500 m). Colored points represent acoustic detections and are sized according to estimated time spent at the receiver station. The shaded area represents the months where few satellite locations were recorded from the SPOT5 tag, and non-shaded areas are months where horizontal locations were frequently obtained from the SPOT tag. The red vertical line indicates the last recorded transmission from the SPOT5 tag

The track of a mature female (M13) equipped with both a SPOT and acoustic tag spanned 366 days and 527 days, respectively. The acoustic tag transmitted data for an additional four months after the SPOT tag stopped transmitting. Seasonality was evident throughout the track (Fig. 5), with a reduction in the number of Argos positions in February, March, and April of 2013. The individual, however, was detected frequently on the DMNP array throughout this period (Fig. 5).

**Fig. 6:**
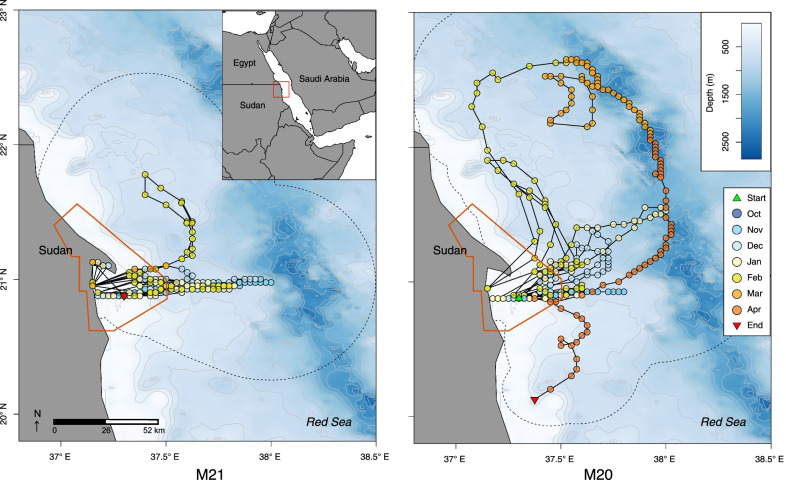
**Left** Movement of an adult female *Mobula alfredi* (M21) and a **Right** mature male *M.* alfredi (M20) equipped with both a MK10AF-satellite and an acoustic tag. Satellite tracks were estimated from light geolocation positioning processed through GPE-3 with incorporated known acoustic COA locations. The orange lines indicate the boundaries of the Dungonab Bay MPA. The dotted lines represent the outer edges of each estimated error ellipse that was calculated for every location point. Release locations are indicated by the red triangle

One MK10AF tag did not report, while the resulting tracks from individuals M20 and M21 were primarily derived from light geolocation positions; M21 recorded no Fastloc-GPS or Argos positions and M20 reported three Argos locations and two Fastloc-GPS points. Both individuals were captured and tagged on October 31st, 2012 near the central part of the array in DMNP and the estimated tracks indicate that they departed Dungonab Bay shortly after tagging and moved offshore to the east. The most likely track for M21 included multiple excursions outside of DMNP (up to 80 km away) over the 124-day monitoring period (Fig. 6). The most likely track for M20 shows a 197 km northward excursion in mid-March before returning briefly to Dungonab Bay and then continuing to move south where the tag detached 84 km from the Bay on April 30th, 2013, after a 182-day track period (Fig. 6).

A single detection of an adult male (M25) was recorded on February 19th, 2013 at Sanganeb Atoll, a submerged reef located in deep water (dropping off from near surface to ~ 400 m depth and ~ 20 km from the coast) 125 km south of DMNP. This individual was last detected in the DMNP array on January 7th, 2013 and next detected on February 21st, 2013. A further two detections were recorded for an immature female (M10) at Suedi Pass on February 2nd, 2014 and a single detection recorded on the August 18th, 2014. This individual was also detected in the DMNP array on February 4th, 2014 and August 16th, 2014. The time interval between detections for these sites indicate the two individuals must have traveled at a minimum rate of 50 km/day. Lastly, a single detection of an adult male (M20) was recorded at Suedi Pass, ~ 70 km south of DMNP on December 10th, 2013.

## Discussion

### Presence and residence

*Mobula alfredi* presence within DMNP was nearly constant, with at least one tagged animal detected on 96% of monitored days. While each individual had extended periods of acoustic absence (21–148 days between detections), the overall RI at DMNP (0.39) was high, especially when compared to other coastal aggregations in Saudi Arabia (RI = 0.24; [38]), Australia (0.14; [17]) and Mozambique (0.16; [60]). In fact, residence at DMNP was more similar to aggregations associated with offshore archipelagos including Chagos (0.39; [19]), and Hawaii (0.39; [61]), though not as high as the Seychelles (0.60; [18]). Higher residency indices associated with offshore archipelagos may be explained by the geographical limitations imposed by these locations’ isolation and lack of continuous coastlines that may aid in discouraging wider ranging behavior. However, the acoustic array design and number of receivers likely plays a significant role in determining the residence index of the studied population, and caution should be used when making comparisons between different regional cohorts.

The two acoustic telemetry datasets on separate *M. alfredi* populations occupying the central Red Sea region provides a unique comparison between two aggregations that inhabit coastlines with dissimilar features. For eight individuals tagged off Al Lith, Saudi Arabia and detected by 65 receivers spread over a much larger area, the RI value was 0.24 [38]. In contrast with Dungonab, *M. alfredi* in Al Lith do not appear to form large aggregations but are usually encountered individually or in small groups of 2–5 individuals (JEMC, MLB, CDB personal observations). In DMNP, the existence of a shallow reef area and large embayment likely provides an environment with predictable food resources and protection, which in turn drives the formation of aggregations and higher residency. Large aggregations of *M. alfredi* have been frequently observed in shallow reef areas such as Hanifaru Bay in the Maldives and Manta Ridge in Raja Ampat, Indonesia [23, 62]. A lack of closely connected functional habitats or reduced embayment at the Saudi Arabian site may explain the different behaviors of its *M. alfredi* cohort. The distinctive structure of the habitats corresponding to differences in behavior suggest that *M. alfredi* home range, site fidelity, and movement patterns are phenotypically plastic responses to local conditions and habitats.

Despite year-round manta presence within DMNP, detection probabilities exhibited a seasonal pattern. The probability of detecting a reef manta ray on any given day increased during the summer, peaked during the fall, and declined through winter. This is consistent with previous visual surveys from the area which reported high numbers of feeding *M. alfredi* in central Dungonab during June, October, and November [30, 41], but no sightings in January or February [40]. Seasonal patterns of *M. alfredi* aggregation and dispersal are common at other sites [16–21, 52], and are often linked to fluctuations in local productivity [21, 63]. This is likely the case at DMNP where peaks in remotely sensed chlorophyll-*a* concentrations are strongly correlated with high detection probabilities (Fig. 2). Generally, coastal waters in the central and southern Red Sea experience high chlorophyll concentrations in the summer that decline through the fall [64], which correlates with the peak season for *M. alfredi* aggregations in DMNP and the corresponding lag in zooplankton productivity that typically follows high chlorophyll-*a* concentrations. The weak but positive correlation between lunar illumination and *M. alfredi* presence also suggests that the area is used as a feeding ground. The lunar cycle’s influence on tidal range, current strength, and food availability could be a contributor to the detection patterns of *M. alfredi* [65–68] and the species has been observed to increase foraging behavior during new and full moon phases [63]. The low percentage of the total variability explained by the chosen GAMM model indicates that unmodeled factors (e.g. tidal flux, submesoscale fronts, small-scale currents around the reefs) may be influencing animal behavior at this site.

*Mobula alfredi* were most likely to be detected from sunrise to mid-morning, peaking around 08:00 in the morning and 15:00 in the afternoon, and least likely to be detected in the first hours after sunset. Diel shifts in habitat use are common among elasmobranchs [69–71] and diurnal dominant detections of *M. alfredi* have been recorded at acoustic arrays in Indonesia, Australia, the Seychelles, and Chagos [16–19, 52]. Increased detections during daytime in this and other acoustic telemetry studies supports reverse diel vertical migration behavior in this species, where individuals generally associate with shallower reefs during the day and move deeper at night to feed on ascending zooplankton. This diel contrast in acoustic detections further corroborates vertical movement data from satellite archival tags that show *M. alfredi* in the Saudi Arabian Red Sea primarily occupying the upper 10 m during daylight hours and making regular excursions to 50 m depth at night [69]. Daytime use of shallow habitats may also be explained by thermoregulatory basking [23], however this is unlikely to be the case in the Red Sea where water temperatures are above 20 C° to 2000 m in depth [72]. It is possible that the heightened levels of reef noise that occurs at night may have obscured signals from the acoustic tags, which would explain the difference in magnitude between nightly and daily detections [44]. Expanded acoustic monitoring of deeper areas or retrieval of archived depth data from depth sensor equipped tags will be necessary to authenticate the observed diel patterns of presence in the DMNP acoustic array.

Residency behavior and habitat selection within Dungonab were not affected by the biological characteristics of tagged *M. alfredi*. There was no significant difference between the RI of males and females and our modeling suggested size and sex were not influential drivers of the observed habitat use. These results are similar to findings from Mozambique [60] but differ slightly from the Seychelles where larger *M. alfredi* had significantly lower RIs [18]. The low sample size of immature *M. alfredi* complicate conclusions regarding the ontogeny of residency behavior; however, the recorded presence of neonates [42] and results from the current study indicate that DMNP is an important site for all life-stages.

### Localized movements within the array

Seasonal trends in detection probability of *M. alfredi* within Dungonab may result from corresponding shifts in manta ray habitat use within the Bay. During the peak aggregation season from late summer through fall, tagged individuals spent most of their time in the central portion of the array where receiver coverage was densest. Detection probability declined in the winter as *M. alfredi* moved north in the Bay where only a single receiver station (N1) was deployed. The high number of winter detections on this single northern receiver in addition to satellite tag locations [30] suggests a seasonal pattern in fine-scale habitat selection within Dungonab. Similar fine-scale seasonal shifts in habitat use have been documented in a whale shark aggregation near Mafia Island, Tanzania which is likely motivated by shifting prey patches within Kilindoni Bay [73]. The exact motivation for the northward shift in *M. alfredi* presence in Dungonab Bay remains unknown but warrants further investigation.

*Mobula alfredi* appeared to move as a cohort throughout the DMNP array in predictable patterns in both years. Spatial networks indicated high levels of movement between the internal main channel exit at C2 and station W2, where movements were twice as frequent as movements between any other receiver pair. Edges were strongest between receivers within the channel and weakest between the channel entrance and receivers to the south, indicating that *M. alfredi* use the channel as a movement corridor. Additionally, mantas were detected less at the receiver in the center of the channel (CH3) than at receivers at the exit and entrance to the channel, potentially indicating transitory behavior as the animals moved into and out of the receiver’s detection range relatively quickly. Fine-scale oceanographic processes such as currents or tides may concentrate zooplankton at the entrance to the channel (CH1, CH2), resulting in focused foraging behavior [74], while the interior of the channel (CH3) may act as a corridor to known cleaning or other feeding sites in the central and western part of the array (C2, C3, W2, NEH personal observation). In situ behavioral observations are needed to confirm these patterns, although these trends have been similarly observed in *M. alfredi* at lagoons elsewhere [14].

### Large-scale excursions from Dungonab

Results from the SPOT-acoustic tagged individual demonstrate the advantages provided by a dual-tagging approach. While few Argos locations were obtained for this individual between late January and mid-April, it was frequently detected on the acoustic array at station N1. Given the high number of Argos derived locations recorded during the months when *M. alfredi* are commonly observed feeding at the surface, the contrasting finding of high numbers of passive acoustic detections and lack of Argos locations from February to April suggests this individual was present in the Bay but spending less time at the surface. This may have been motivated by reduced surface foraging opportunities driven by changes in zooplankton abundance or distribution. Seasonal changes in vertical behavior in response to vertically shifting zooplankton prey have been recorded in the oceanic manta ray (*Mobula birostris*), where surface occupancy in boreal winter shifts to depths of 100–150 m during the boreal summer [75]. It is unclear whether similar seasonal patterns in vertical distribution were responsible for the reduction in Argos detections for the SPOT tagged individual in Dungonab.

The multi-tagging approach used in this study also allowed the observation of movements outside the boundaries of the MPA where acoustic receiver coverage was not feasible. However, movements derived from geolocation PSAT data should be interpreted with caution. Previous work examining the accuracy of light-based geolocation, including with the GPE3 model [39], suggests mean error is typically on the order of ~ 1°. While the light-based geolocations for one tagged *M. alfredi* indicated a northern excursion of 197 km, the lack of GPS data during this time leads to large-scale uncertainty in these pointwise position estimates. However, the few Argos locations near the end of deployment and subsequent release location demonstrate that this individual moved at least 84 km south of the array at the end of April 2013. In addition, acoustic telemetry profiles of two individuals detected at Suedi Pass and Sanganeb Atoll demonstrate that these individuals must have moved a minimum of 50 km per day between the DNMP array and these sites, indicating high mobility consistent with previous studies at other aggregations in the Red Sea [38] and Indian Ocean [18, 19]. These few offshore detections, in addition to the GAMMs, KUDs, and satellite tracks, suggest seasonal dispersal activity and connectivity to the central Sudanese coast. While these data (coupled with the disappearance of four individuals from the DMNP array) indicate Dungonab *M. alfredi* are capable of widespread dispersal as observed elsewhere [11, 12], 15 of 19 acoustically tracked individuals displayed high site fidelity, with detections occurring after prolonged absences.

### Management implications

Dungonab Bay is a critical habitat for *M. alfredi* in the Red Sea and should be managed as a sustainable natural resource for the people of Sudan. Several conservation policies could help mitigate negative human impacts at the site, including seasonal boating speed limits at known *M. alfredi* hotspots [73, 76], regulating the use of gillnet fishing in Dungonab Bay, and introducing codes of conduct for ecotourism operators [77, 78]. The year-round presence and predictable, annual peak aggregation of *M. alfredi* that occurs in the fall means that reliable encounters can be marketed towards tourists seeking to observe these animals in the wild. Although geopolitical instability in Sudan may limit efforts to develop land-based ecotourism in Dungonab, Sudan has possessed a stable liveaboard diving industry in the region for decades and several of these operators seasonally visit Dungonab for *M. alfredi* snorkeling excursions. A responsible expansion of these expeditions in Dungonab could provide income for local communities and financial incentives for the continued protection and conservation of the species at this site.

Successful protection of *M. alfredi* at Dungonab could have far reaching benefits. Due to the migratory capabilities of this species (at least 1150 km along continuous coastline habitat) [11], DMNP could represent a source of stock replenishment for depleted sites elsewhere. Although direct exchange between Dungonab Bay and the next closest aggregation in Saudi Arabia has not been documented, genetic analysis could quantify the degree of connectivity between these two sites and to other populations in the Western Indian Ocean. The movements of three individuals to Suedi Pass and Sanganeb Atoll suggest a regional rather than a localized management plan is required in Sudan. These sites are all within the buffer zone that encompasses the serial World Heritage Site [43], providing a mechanism by which such a management strategy could be applied, though enforcement remains a problem.

### Tagging methodology

A live capture technique for manta rays was developed in the current study given the challenges of effectively placing external tags on free swimming animals (i.e. proper placement) and reported shedding rates that have limited long-term studies on this species. We adopted and modified standard capture, tag, and release methodologies commonly applied in the study of elasmobranchs [79]. This is the first study to surgically implant internal acoustic tags and directly attach satellite tags (SPOT) to the dorsal fin of *M. alfredi*. Our technique was efficient for capturing animals. While individuals demonstrated an initial escape response following hooking, including increased swim speed and directed straight line movement, individuals quickly settled down and the floats allowed time for the animal to swim freely and tire for ease of handling. Monitoring the float movements following captures, such as rapid directional movement at surface versus slow more tortuous movement, provided a good indicator of when the animal was in an appropriate state for handling. The benefits of internal implantation include longer tag retention and reduced biofouling when compared to externally placed tags. The latter is an important consideration given the demonstrated impacts of externally placed tags on elasmobranchs, such as tissue damage, increased weight burden that can impair movement, reduced growth rates, and potential for non-natural species interactions [80–82].

The current study using internally placed tags reported high tag retention and consistency across the tagged cohort, with 15 out of 20 mantas tracked for nearly two years (mean 599 ± 172.4 SD track days for 20 tagged individuals) contrasting previous tagging studies that reported an average of 284 ± 187 SD track days with 33 tagged animals [18] and 118 ± 18 SD track days for 42 tagged individuals [60]. However, it should be noted that long-term tag retention for externally placed acoustic tags on *M. alfredi* has been successful and observed track days of up to 1,555 days have been reported in Chagos (mean 585 ± 514 SD track days, [19]). The study in Dungonab was designed to run for five years but unfortunately was terminated due to unforeseen changes in the geopolitical situation in Sudan. This limited our ability to quantify the increased tag retention associated with internal tagging on this species beyond the time series presented here. In terms of the SPOT tag placement, we adopted identical approaches for attachment used for other elasmobranchs [83]. This led to retention of tags for periods of up to 366 days with a mean of 207.7 ± 160 SD days for all three animals fitted with SPOT tags during the October 2012 field expedition [30], which is considerably longer that tracks previously reported (mean 27 ± 21.6 SD days, [84]; mean 62 ± 31.9 SD days [12]). Previous work assessing the impact of direct attachment of SPOT tags to the dorsal fins of sharks have also identified limited impact [85, 86].

We recognize that disadvantages associated with the live capture method include an increased initial stress response tied t o the capture/tagging process. Capture and handling stress can alter animal behavior in the short term and some elasmobranchs can exhibit long-term impacts [87]. Susceptibility to capture stress varies widely by species, and its effects on *M. alfredi* and other Mobulids are understudied. However, the long-term tracking of the tagged mantas (two years of near continuous data) indicate that survivorship was high and animals displayed normal behavior. In addition, several individuals were re-sighted within 24 h of the capture-handling-tagging procedures engaged in what appeared to be normal feeding and aggregation behavior (i.e. tagged individuals observed in chains of mantas; NEH personal observation). As with any invasive procedure tied to electronic tracking of animals, the costs and benefits need to be assessed within the context of the species and the study question. Given the data generated and the response of the tagged animals, we suggest the adopted approach provides an appropriate method for future studies where long-term monitoring, accurate demographic data, and/or more thorough biosampling (blood, muscle, etc.), of individuals is a necessity. This is important given the need for long-term time series data to truly understand animal movement ecology [83] and our limited understanding of long-term movements of *M. alfredi*.

## Conclusions

Two years of passive acoustic data indicate *M. alfredi* demonstrate high residence in and site fidelity to Dungonab Bay. Comparing detection counts among receivers revealed seasonal patterns of *M. alfredi* habitat selection within the DMNP array and suggested environmental drivers of *M. alfredi* presence. For individuals equipped with multiple tags, satellite telemetry geolocation data incorporating known acoustic positions revealed larger scale movements of two individuals that exhibited multiple excursions from and returns to DMNP. These results largely confirm previous visual survey data [40–42] and provide an in-depth description of *M. alfredi* movement ecology across Dungonab Bay. These baseline data could be instrumental in directing future research, implementing conservation actions, and for assisting the development of sustainable ecotourism in this region.

## Supplementary Information

**Additional file 1: Fig. S1**. Map of acoustic receivers located outside of Dungonab Bay at Suedi Pass and Sanganeb Atoll.

**Additional file 2: Table S1**. GAMM AIC values for all tested models listed from lowest to highest ΔAIC values. All models included both biological variables and the number of stations in addition to *Mobula alfredi* identity as a random effect.

## Data Availability

The datasets used and/or analyzed during the current study are available from the corresponding author on reasonable request.
