# Correction: Home sweet home: spatiotemporal distribution and site fidelity of the reef manta ray (*Mobula alfredi*) in Dungonab Bay, Sudan

**DOI:** 10.1186/s40462-022-00325-6

**Published:** 2022-05-20

**Authors:** Anna M. Knochel, Nigel E. Hussey, Steven T. Kessel, Camrin D. Braun, Jesse E. M. Cochran, Graham Hill, Rebecca Klaus, Tarik Checkchak, Nasereldin M. Elamin El Hassen, Mohammed Younnis, Michael L. Berumen

**Affiliations:** 1 Red Sea Research Center, Division of Biological and Environmental Science and Engineering, King Abdullah University of Science and Technology, Thuwal, 23955 Kingdom of Saudi Arabia; 2 Department of Integrative Biology, University of Windsor, 401 Sunset Avenue, Windsor, ON Canada; 3 Equipe Cousteau, Paris, France; 4 Daniel P. Haerther Center for Conservation and Research, John G. Shedd Aquarium, Chicago, IL 60605 USA; 5 Biology Department, Woods Hole Oceanographic Institution, Woods Hole, MA 02543 USA; 6 The Deep Aquarium, Hull, UK; 7 Wildlife Conservation General Administration, Port Sudan, Sudan

## Correction to: Movement Ecology (2022) 10:22. https://doi.org/10.1186/s40462-022-00314-9.

Following publication of the original article [1], it was noted that due to a typesetting error, a duplicated version of Figure 6 was published as Figure 5. The correct Figure 5 has been included in this Correction and the original article has been corrected.

**Fig. 5:**
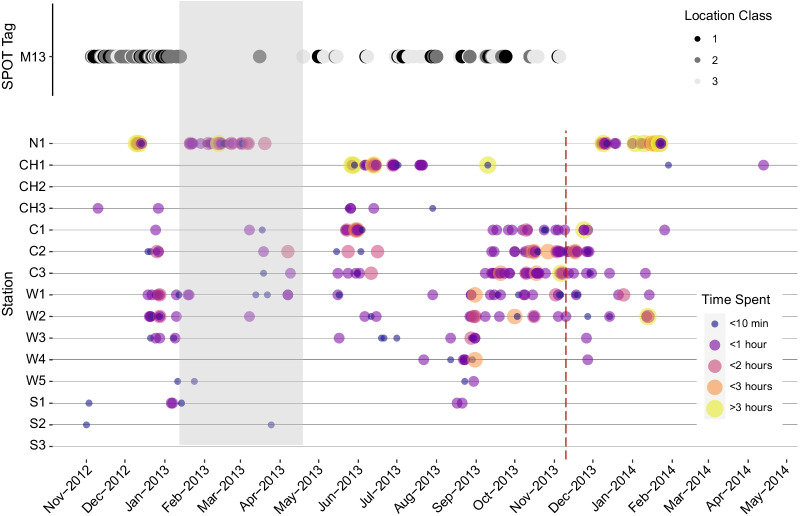
Spatial–temporal residency duration plot for an adult female *Mobula alfredi* (M13), equipped with both a SPOT and acoustic tag. Greyscale points represent transmissions from the SPOT5 tag and their estimated accuracy errors (3: < 250 m; 2: 250 to < 500 m; 1: 500 to < 1500 m). Colored points represent acoustic detections and are sized according to estimated time spent at the receiver station. The shaded area represents the months where few satellite locations were recorded from the SPOT5 tag, and non-shaded areas are months where horizontal locations were frequently obtained from the SPOT tag. The red vertical line indicates the last recorded transmission from the SPOT5 tag

The publisher apologises to the authors and readers for the inconvenience caused by the error.
