# Supplementary figures and images for: Home sweet home: spatiotemporal distribution and site fidelity of the reef manta ray (Mobula alfredi) in Dungonab Bay, Sudan
Source: Mov Ecol. 2022 Apr 28;10:22. doi: 10.1186/s40462-022-00314-9 (PMC9052681; doi:10.1186/s40462-022-00314-9)

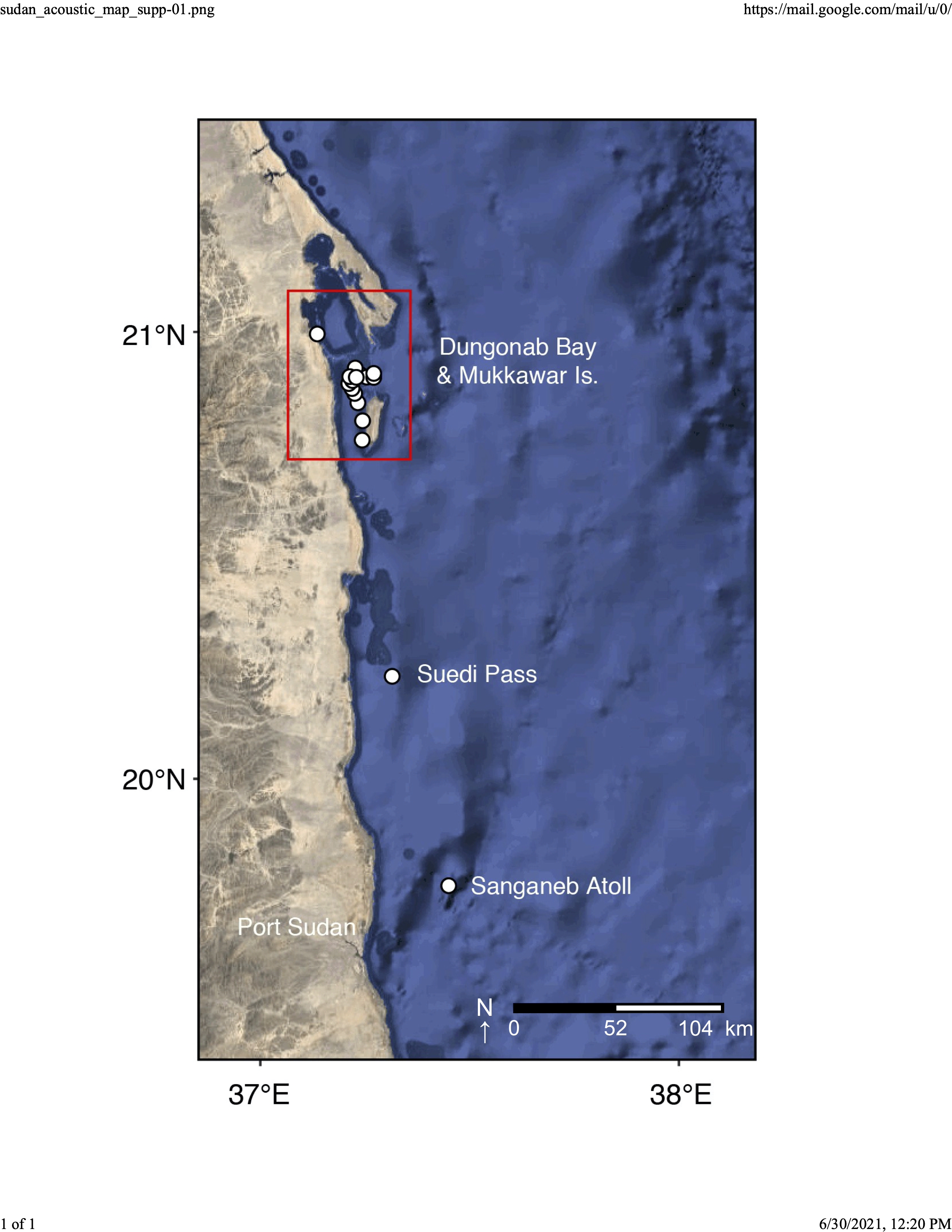

Supplement: Supplementary file 1 — Additional file 1: Fig. S1. Map of acoustic receivers located outside of Dungonab Bay at Suedi Pass and Sanganeb Atoll. [file 40462_2022_314_MOESM1_ESM.jpg]
