# Supplementary material for: Home sweet home: spatiotemporal distribution and site fidelity of the reef manta ray (Mobula alfredi) in Dungonab Bay, Sudan
Source: Mov Ecol. 2022 Apr 28;10:22. doi: 10.1186/s40462-022-00314-9 (PMC9052681; doi:10.1186/s40462-022-00314-9)
Supplement: Supplementary file 2 — Additional file 2: Table S1. GAMM AIC values for all tested models listed from lowest to highest ΔAIC values. All models included both biological variables and the number of stations in addition to Mobula alfredi identity as a random effect. [file 40462_2022_314_MOESM2_ESM.pdf]

Table S1.

**Model**	**AIC**	**△ AIC**
Presence ~ Day + Hour + chla + moon	134134.3	0
Presence ~ Day + Hour + chla	134216.8	82.4
Presence ~ Day + Hour + moon	134707.8	573.5
Presence ~ Day + Hour	134830.4	696.1
Presence ~ Day + chla + moon	135299.9	1159.5
Presence ~ Day + chla	135381.9	1165.6
Presence ~ Day + moon	135870.3	1736.0
Presence ~ Day	135992.2	1857.9
Presence ~ Hour + chla + moon	140563.4	6429.1
Presence ~ Hour + chla	140589.1	6454.8
Presence ~ moon + chla	141697.9	7563.6
Presence ~ chla	141723.5	7589.2
Presence ~ Hour + moon	142185.4	8051.1
Presence ~ Hour	142299.0	8164.7
Presence ~ moon	143312.9	9178.6
Presence ~ 1	143425.9	9291.6
